# Preparation, Structure, and Bioactivities of Medicinal and Edible Homologous Plant Polysaccharides: A Review

**DOI:** 10.1002/fsn3.70453

**Published:** 2025-08-05

**Authors:** Mengnan Chen, Wenfeng Wei, Zhuang Wang, Weiming Wang

**Affiliations:** ^1^ Heilongjiang Academy of Traditional Chinese Medicine Harbin China

**Keywords:** biological activities, extraction and purification, medicinal and edible homologous, plant polysaccharides, structure elucidation

## Abstract

As of 2023, the National Health Commission has announced 102 MEHs varieties, with 94 being plant‐erived. Their development and application show great potential. The plant polysaccharides (MEHPPs) are one category of the main and representative pharmacologically active macromolecules in MEH that have many biological activities both in vitro and in vivo, such as immune regulation, antioxidant, hypoglycemic, lipid‐lowering, antitumor, anti‐inflammatory, etc. In addition, the quality of MEHPPs obtained from different raw materials via sundry extraction and purification methods is different. At the same time, the structural analysis of MEHPPs is the focus of studying the molecular weight determination, monosaccharide composition, glucoside linkage mode, and partial conformation. Based on the literature search, this article reviews the preparation methods, structural characterization, biological activities, and potential mechanisms of MEHPPs. It provides a basis for the development of safe and effective health care products and new drugs for the prevention and treatment of many chronic diseases.

## Graphical abstract

This article reviews the research progress of medicinal and edible homologous plant polysaccharides (MEHPPs). It introduces their extraction and purification methods, expounds on structural characterization techniques, and elaborates on various biological activities and their mechanisms, providing a basis for the development of drugs and health products for the prevention and treatment of related chronic diseases.

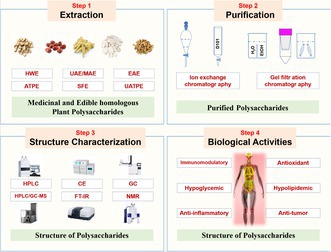

**Abbreviations**
^2^D‐NMR: two‐dimensional nuclear magnetic resonance
ACE: acid extraction
ALE: alkali extraction
CE: capillary electrophoresis
DPV‐ALLS: differential pressure viscosity‐angle laser light scattering detector
EAE: enzyme‐assisted extraction
ELSD: evaporative light scattering detector
FT‐IR: Fourier transform infrared spectroscopy
GC: gas chromatography
GC–MS: gas chromatography–mass spectrometry
HPGFC: highperformance gel filtration chromatography
HPGPC: highperformance gel permeation chromatography
HPLC: highperformance liquid chromatography
HPSEC: highperformance size exclusion chromatography
HWE: hot water extraction
LC–MS: liquid chromatography—mass spectrometry
MAE: microwave‐assisted extraction
MALLS: multi‐angle laser light scattering detector
MEHP: medicinal and edible homologous plant
MEHPPs: medicinal and edible homologous plant polysaccharides
MEHs: medicinal and edible homologous
RID: Refractive Index Detector
SFE: supercritical CO_2_ fluid extraction
UAE: ultrasonic‐assisted extraction

## 1 Introduction

With the improvement of the economy, people begin to pay more attention to their health and diet quality. In recent years, various health care products developed from the concept of “medicinal and edible homologous (MEH)” in The Yellow Emperor's Canon of Internal Medicine, have become more and more popular (Xu et al. 2022). In traditional Chinese culinary culture, Chinese medicinal herbs are widely consumed as ingredients in the folk. According to the Chinese Food Safety Law, drugs are not allowed to be added to food produced and marketed, but substances belonging to MEH may be added. In Feb. 2002, the former Health Department promulgated 86 kinds of dual use of medicine and food TCM; In Jan. 2020, 6 kinds of TCM were added into MEH directories, including *Angelica sinensis*, *Rhizoma Kaempferiae*, *Croci Stigma*, etc.; In Nov. 2023, 9 new MEH plants were released, such as *Codonopsis pilosula* , *Panax quinquefolius* , *Astragalus membranaceus*, etc. In total, 102 kinds of MEH varieties had been announced by the National Health Commission as of now. According to the different sources of raw materials, they can be grouped into three categories: herbal medicine (94), animal medicine (6), and fungal medicine (2), showing in Table 1 for details. As one of the most important sources of traditional Chinese medicine, plants have a long medicinal history in the prevention and treatment of human diseases, and occupy a major position in the natural MEH (Xue, Wang, et al. 2022).

**TABLE 1:** Detailed information of 103 MEHs.

No.	Name of substance	Latin name of raw materials	Source of family	Officinal parts	Year of promulgation	Categories
1	Dingxiang	*Eugenia caryophyllata* Thunb.	Myrtaceae Juss.	Flower bud	2002	Plant
2	Bajiaohuixiang	*Illicium verum* Hook.f.	Magnoliaceae Juss.	Ripe fruit	2002	Plant
3	Daodou	*Canavalia gladiata* (Jacq.) DC.	Fabaceae Lindl.	Mature seed	2002	Plant
4	Xiaohuixiang	*Foeniculum vulgare* Mill.	Apiaceae Lindl.	Ripe fruit; Leaves and stalks are used for seasoning	2002	Plant
5	Xiaoji	*Cirsium setosum* (Willd.) MB.	Asteraceae Bercht. & J. Presl	Overground part	2002	Plant
6	Shanyao	*Dioscorea opposita* Thunb.	Dioscoreaceae R. Br.	Rhizome	2002	Plant
7	Shanzha	*Crataegus pinnatifida* Bge. var. major N.E.Br.	Rosaceae Juss.	Ripe fruit	2002	Plant Plant
		*Crataegus pinnatifida* Bge.	Rosaceae Juss.			
8	Machixian	*Portulaca oleracea* L.	Portulacaceae Juss.	Overground part	2002	Plant
9	Wumei	*Prunus mume* (Sieb.) Sieb. et Zucc.	Rosaceae Juss.	Near ripe fruit	2002	Plant
10	Mugua	*Chaenomeles speciosa* (Sweet) Nakai	Rosaceae Juss.	Near ripe fruit	2002	Plant
11	Huomaren	*Cannabis sativa* L.	Moraceae Gaudich.	Ripe fruit	2002	Plant
12	Daidaihua	*Citrus aurantium* L. var. amara Engl.	Rutaceae Juss.	Flower bud	2002	Plant
13	Yuzhu	*Polygonatum odoratum* (Mill.) Druce	Liliaceae Juss.	Rhizome	2002	Plant
14	Gancao	*Glycyrrhiza uralensis* Fisch.	Fabaceae Lindl.	Root and rhizome	2002	Plant Plant Plant
		*Glycyrrhiza inflata* Bat.	Fabaceae Lindl.			
		*Glycyrrhiza glabra* L.	Fabaceae Lindl.			
15	Baizhi	*Angelica dahurica* (Fisch. ex Hoffm.) Benth. et Hook. f.	Apiaceae Lindl.	Root	2002	Plant
		*Angelica dahuric*a (Fisch. ex Hoffm.) Benth. et Hook. f. var. *formosana* (Boiss.) Shan et Yuan	Apiaceae Lindl.			
16	Baiguo	*Ginkgo biloba* L.	Ginkgoaceae Engl.	Mature seed	2002	Plant
17	Baibiandou	*Dolichos lablab* L.	Fabaceae Lindl.	Mature seed	2002	Plant
18	Baibiandouhua	*Dolichos lablab* L.	Fabaceae Lindl.	Flower	2002	Plant
19	Longyanrou	*Dimocarpus longan* Lour.	Sapindaceae Juss.	Aril	2002	Plant
20	Juemingzi	*Cassia obtusifolia* L.	Fabaceae Lindl.	Mature seed	2002	Plant
		*Cassia tora* L.	Fabaceae Lindl.			Plant
21	Baihe	*Lilium lancifolium* Thunb.	Liliaceae Juss.	Fleshly scale leaf	2002	Plant
		*Lilium brownie* F.E. Brown var. *viridulum* Baker	Liliaceae Juss.			
		*Lilium pumilum* DC.	Liliaceae Juss.			
22	Roudoukou	*Myristica fragrans* Houtt.	Myristicaceae R. Br.	Kernel and seed coat	2002	Plant
23	Rougui	*Cinnamomum cassia* Presl	Lauraceae Juss.	Bark	2002	Plant
24	Yuganzi	*Phyllanthus emblica* L.	Euphorbiaceae Juss.	Ripe fruit	2002	Plant
25	Foshou	*Citrus medica* L. var. *sarcodactylis* Swingle	Rutaceae Juss.	Fruit	2002	Plant
26	Xingren	*Prunus armeniaca* L. var. *ansu* Maxim	Rosaceae Juss.	Mature seed	2002	Plant
		*Prunus sibirica* L.	Rosaceae Juss.			
		*Prunus mandshurica* (Maxim) Koehne	Rosaceae Juss.			
		*Prunus armeniaca* L.	Rosaceae Juss.			
27	Shaji	*Hippophae rhamnoides* L.	Elaeagnaceae Juss.	Ripe fruit	2002	Plant
28	Qianshi	*Euryale ferox* Salisb.	Nymphaeaceae Salisb.	Mature seed kernel	2002	Plant
29	Huajiao	*Zanthoxylum schinifolium* Sieb. et Zucc.	Rutaceae Juss.	Ripe peel	2002	Plant
		*Zanthoxylum bungeanum* Maxim.	Rutaceae Juss.			
30	Chixiaodou	*Vigna umbellata Ohwi* et Ohashi	Fabaceae Lindl.	Mature seed	2002	Plant
		*Vigna angularis Ohw*i et Ohashi	Fabaceae Lindl.			
31	Maiya	*Hordeum vulgare* L.	Poaceae Barnhart	Processed product of germinated and dried ripe fruit	2002	Plant
32	Kunbu	*Laminaria japonica* Aresch.	Laminariaceae	Thallus	2002	Plant
		Ecklonia kurome Okam.	Alariaceae			
33	Zao	*Ziziphus jujuba* Mill.	Rhamnaceae Juss.	Ripe fruit	2002	Plant
		*Ziziphus jujuba* Mill. var. *spinosa* (Bunge) Hu ex H. F. Chou	Rhamnaceae Juss.	Ripe fruit	2002	Plant
		*Diospyros lotus* L.	Ebenaceae Gürke	Ripe fruit	2002	Plant
34	Luohanguo	*Siraitia grosvenorii* (Swingle.) C.Jeffrey ex A.M. Lu et Z.Y. Zhang	Cucurbitaceae Juss.	Fruit	2002	Plant
35	Yuliren	*Prunus humilis* Bge.	Rosaceae Juss.	Mature seed	2002	Plant
		*Prunus japonica* Thunb.	Rosaceae Juss.			
		*Prunus pedunculata* Maxim.	Rosaceae Juss.			
36	Jinyinhua	*Lonicera japonica* Thunb.	Caprifoliaceae Juss.	Buds or budding Flowers	2002	Plant
37	Qingguo	*Canarium album* Raeusch.	Burseraceae	Ripe fruit	2002	Plant
38	Yuxingcao	*Houttuynia cordata* Thunb.	Saururaceae Rich. ex T. Lestib.	Whole grass or ground parts	2002	Plant
39	Jiang	*Zingiber officinale* Rosc.	Zingiberaceae	Rhizome	2002	Plant
40	Zhijuzi	*Hovenia dulcis* Thunb.	Rhamnaceae Juss.	Mature seeds or fruit with an inflorescence axis	2002	Plant
		*Hovenia acerba* Lindl.	Rhamnaceae Juss.			
		*Hovenia trichocarpa* Chun et Tsiang	Rhamnaceae Juss.			
41	Gouqizi	*Lycium barbarum* L.	Solanaceae Juss.	Ripe fruit	2002	Plant
42	Zhizi	*Gardenia jasminoides* Ellis	Rubiaceae Juss.	Ripe fruit	2002	Plant
43	Sharen	*Amomum villosum* Lour.	Zingiberaceae	Ripe fruit	2002	Plant
		*Amomum villosum* Lour. var. xanthioides T.L.Wu et Senjen	Zingiberaceae			
		Amomum longiligularg T.L.Wu	Zingiberaceae			
44	Pangdahai	*Sterculia lychnophora* Hance	Sterculiaceae	Mature seed	2002	Plant
45	Xiangyuan	*Citrus medica* L.	Rutaceae Juss.	Ripe fruit	2002	Plant
		*Citrus wilsonii* Tanaka	Rutaceae Juss.			
46	Xiangru	*Mosla chinensis* Maxim.	Lamiaceae Martinov	Overground part	2002	Plant
		*Mosla chinensis* ‘jiangxiangru’	Lamiaceae Martinov			
47	Taoren	*Prunus persica* (L.) Batsch	Rosaceae Juss.	Mature seed	2002	Plant
		*Prunus davidiana* (Carr.) Franch.	Rosaceae Juss.			
48	Sangye	*Morus alba* L.	Moraceae Gaudich.	Leaf	2002	Plant
49	Sangshen	*Morus alba* L.	Moraceae Gaudich.	Ripe fruit	2002	Plant
50	Juhong	*Citrus reticulata* Blanco	Rutaceae Juss.	Outer peel	2002	Plant
51	Jiegeng	*Platycodon grandiflorum* (Jacq.) A. DC.	Campanulaceae Juss.	Root	2002	Plant
52	Yizhiren	*Alpinia oxyphylla* Miq.	Zingiberaceae	Ripe fruit	2002	Plant
53	Heye	*Nelumbo nucifera* Gaertn.	Nymphaeaceae Salisb.	Leaf	2002	Plant
54	Laifuzi	*Raphanus sativus* L.	Brassicaceae Burnett	Mature seed	2002	Plant
55	Lianzi	*Nelumbo nucifera* Gaertn.	Nymphaeaceae Salisb.	Mature seed	2002	Plant
56	Gaoliangjiang	*Alpinia officinarum* Hance	Zingiberaceae	Rhizome	2002	Plant
57	Danzhuye	*Lophatherum gracile* Brongn.	Poaceae Barnhart	Stem leaf	2002	Plant
58	Dandouchi	*Glycine max* (L.) Merr.	Fabaceae Lindl.	Mature seeds	2002	Plant
59	Juhua	*Chrysanthemum morifolium* Ramat.	Asteraceae Bercht. & J. Presl	Capitulum	2002	Plant
60	Juju	*Cichorium glandulosum* Boiss. et Huet	Asteraceae Bercht. & J. Presl	Overground part or root	2002	Plant
		*Cichorium intybus* L.	Asteraceae Bercht. & J. Presl			
61	Huangjiezi	*Brassica juncea* (L.) Czern. et Coss	Brassicaceae Burnett	Mature seed	2002	Plant
62	Huangjing	*Polygonatum kingianum* Coll. et Hemsl.	Liliaceae Juss.	Rhizome	2002	Plant
		*Polygonatum sibiricum* Red.	Liliaceae Juss.			
		*Polygonatum cyrtonema* Hua	Liliaceae Juss.			
63	Zisu	*Perilla frutescens* (L.) Britt.	Lamiaceae Martinov	Leaf and Twig	2002	Plant
64	Zisuzi	*Perilla frutescens* (L.) Britt.	Lamiaceae Martinov	Ripe fruit	2002	Plant
65	Gegen	*Pueraria lobata* (Willd.) Ohwi	Fabaceae Lindl.	Root	2002	Plant
66	Heizhima	*Sesamum indicum* L.	Pedaliaceae	Mature seed	2002	Plant
67	Heihujiao	*Piper nigrum* L.	Piperaceae Giseke	Near ripe or ripe fruit	2002	Plant
68	Huaimi	*Sophora japonica* L.	Fabaceae Lindl.	Flower bud	2002	Plant
69	Huaihua	*Sophora japonica* L.	Fabaceae Lindl.	Flower	2002	Plant
70	Pugongying	*Taraxacum mongolicum* Hand‐Mazz.	Asteraceae Bercht. & J. Presl	Whole herb	2002	Plant
		*Taraxacum borealisinense* Kitam.	Asteraceae Bercht. & J. Presl			
71	Feizi	*Torreya grandis* Fort.	Taxaceae Gray	Mature seed	2002	Plant
72	Suanzaoren	*Ziziphus jujuba* Mill. var. *spinosa* (Bunge) Hu ex H.F.Chou	Rhamnaceae Juss.	Mature seeds	2002	Plant
73	Xianbaimaogen	*Imperata cylindrica*l Beauv. var. *major* (Nees) C.E. Hubb.	Poaceae Barnhart	Rhizome	2002	Plant
74	Xianlugen	*Phragmites communis* Trin.	Poaceae Barnhart	Rhizome	2002	Plant
75	Jupi	*Citrus reticulata* Blanco	Rutaceae Juss.	Ripe peel	2002	Plant
76	Bohe	*Mentha haplocalyx* Briq.	Lamiaceae Martinov	Overground part	2002	Plant
77	Yiyiren	*Coix lacryma‐jobi* L. var. *mayuen*. (Roman.) Stapf	Poaceae Barnhart	Mature seed kernel	2002	Plant
78	Xiebai	*Allium macrostemon* Bge.	Liliaceae Juss.	Bulb	2002	Plant
		*Allium chinense* G. Don	Liliaceae Juss.			
79	Fupenzi	*Rubus chingii* Hu	Rosaceae Juss.	Fruit	2002	Plant
80	Huoxiang	*Pogostemon cablin* (Blanco) Benth.	Lamiaceae Martinov	Overground part	2002	Plant
81	Danggui	*Angelica sinensis* (Oliv.) Diels.	Apiaceae Lindl.	Root	2020	Plant
82	Shannai	*Kaempferia galanga* L.	Zingiberaceae	Rhizome	2020	Plant
83	Xihonghua	*Crocus sativus* L.	Iridaceae Juss.	Stigma	2020	Plant
84	Caoguo	*Amomum tsao‐ko* Crevost et Lemaire	Zingiberaceae	Ripe fruit	2020	Plant
85	Jianghuang	*Curcuma Longa* L.	Zingiberaceae	Rhizome	2020	Plant
86	Biba	*Piper longum* L.	Piperaceae Giseke	Ripe or nearly ripe ear	2020	Plant
87	Dangshen	*Codonopsis pilosula* (Franch.) Nannf.	Campanulaceae Juss.	Root	2023	Plant
		*Codonopsis pilosula* Nannf. var. *modesta* (Nannf.) L.T. Shen				
		*Codonopsis tangshen* Oliv.				
88	Roucongrong	*Cistanche deserticola* Y.C. Ma	Orobanchaceae Vent.	Succulent stem	2023	Plant
89	Tiepishihu	*Dendrobium officinale* Kimuraet Migo	Orchidaceae Juss.	Stem	2023	Plant
90	Xiyangshen	*Panax quinquefolium* L.	Araliaceae Juss.	Root	2023	Plant
91	Huangqi	*Astragalus membranaceus* (Fisch.) Bge. var. *mongholicus* (Bge.) Hsiao	Fabaceae Lindl.	Root	2023	Plant
92	Shanzhuyu	*Cornus officinalis* Sieb. et Zucc.	Cornaceae Bercht. & J. Presl	Fruit	2023	Plant
93	Tianma	*Gastrodia elata* B1.	Orchidaceae Juss.	Tuber	2023	Plant
94	Duzhongye	*Eucommia ulmoides* Oliv.	Eucommiaceae Engl.	Leaf	2023	Plant
95	Wushaoshe	*Zaocys dhumnades* (Cantor)	Colubridae	Peel and remove the whole viscera	2002	Animal
96	Muli	*Ostrea gigas* Thumberg	Ostreidae	Conch	2002	Animal
		*Ostrea talienwhanensis* Crosse	Ostreidae			
		*Ostrea rivularis* Gould	Ostreidae			
97	Ejiao	*Equus asinus* L.	Equidae	A solid glue made from dried or fresh skin by decocting and concentrating	2002	Animal
98	Jineijin	*Callus gallus domesticus* Brisson	Phasianidae	Inner wall of chicken sandbag	2002	Animal
99	Fengmi	*Apis cerena* Fabricius	Apidae	Honey made by bees	2002	Animal
100	Fushe	*Agkistrodon halys* (Pallas)	Viperidae	Peel and remove the whole viscera	2002	Animal
101	Fuling	*Poria cocos* (Schw.) Wolf	Polyporaceae	Sclerotium	2002	Fungal
102	Lingzhi	*Ganoderma lucidum* (Leyss. ex Fr.) Karst.	Polyporaceae	Polysaccharide	2023	Fungal
		*Ganoderma sinense* Zhao, Xu et Zhang	Polyporaceae			

In recent years, the active components of herbal medicines have become a research hotspot in the field of natural products. More and more active ingredients have been isolated and identified from MEH, including polysaccharides, flavonoids, alkaloids, volatile oils, terpenoids, saponins, proteins, and vitamins (Li et al. 2022). Polysaccharides are a class of water‐soluble and polar metabolites existing in plants. The therapeutic potential of natural plant polysaccharides isolated and identified from MEHs plants (MEHPPs) for some chronic and difficult diseases has attracted extensive attention worldwide. Previous studies had shown that the content of MEHPPs was very low in plants, and their activity was greatly affected by extraction methods. Therefore, researchers have proposed some novel and efficient methods for the extraction and purification of MEHPPs (Zhi et al. 2024). Advances in analytical techniques have greatly facilitated the overall characterization of MEHPPs at multiple levels. Due to the large molecular weight, polarity, and lack of chromophore of polysaccharides, multi–angle laser light scattering detector (MALLS) (Zhu et al. 2022), charged Aerosol Detector (CAD) (Corrado et al. 2023), evaporative light scattering detector (ELSD) (Cheng et al. 2021), refractive index detector (RID) and MS are widely applied to the online detection of polysaccharides and hydrolyzed products (Liu et al. 2022; Cheong et al. 2015). MEHPPs are polymerized from more than 10 monosaccharides, and the types, arrangement, binding mode, and spatial conformation are related to the biological activity of polysaccharide. Therefore, it is important to characterize the primary structure and advanced structure of MEHPPs, which will help to fully understand the structure–activity relationship of MEHPPs. Furthermore, phytochemical and biological studies have confirmed that MEHPPs obtained by various extraction and purification methods, as an important bioactive component of MEH Chinese medicine, are specifically involved in the regulation of various key functions, including immunomodulatory, antioxidant, hypoglycemic, hypolipidemic, antitumor, anti‐inflammatory, and other activities due to its special structure (Xue, Li, et al. 2022).

**FIGURE 1:**
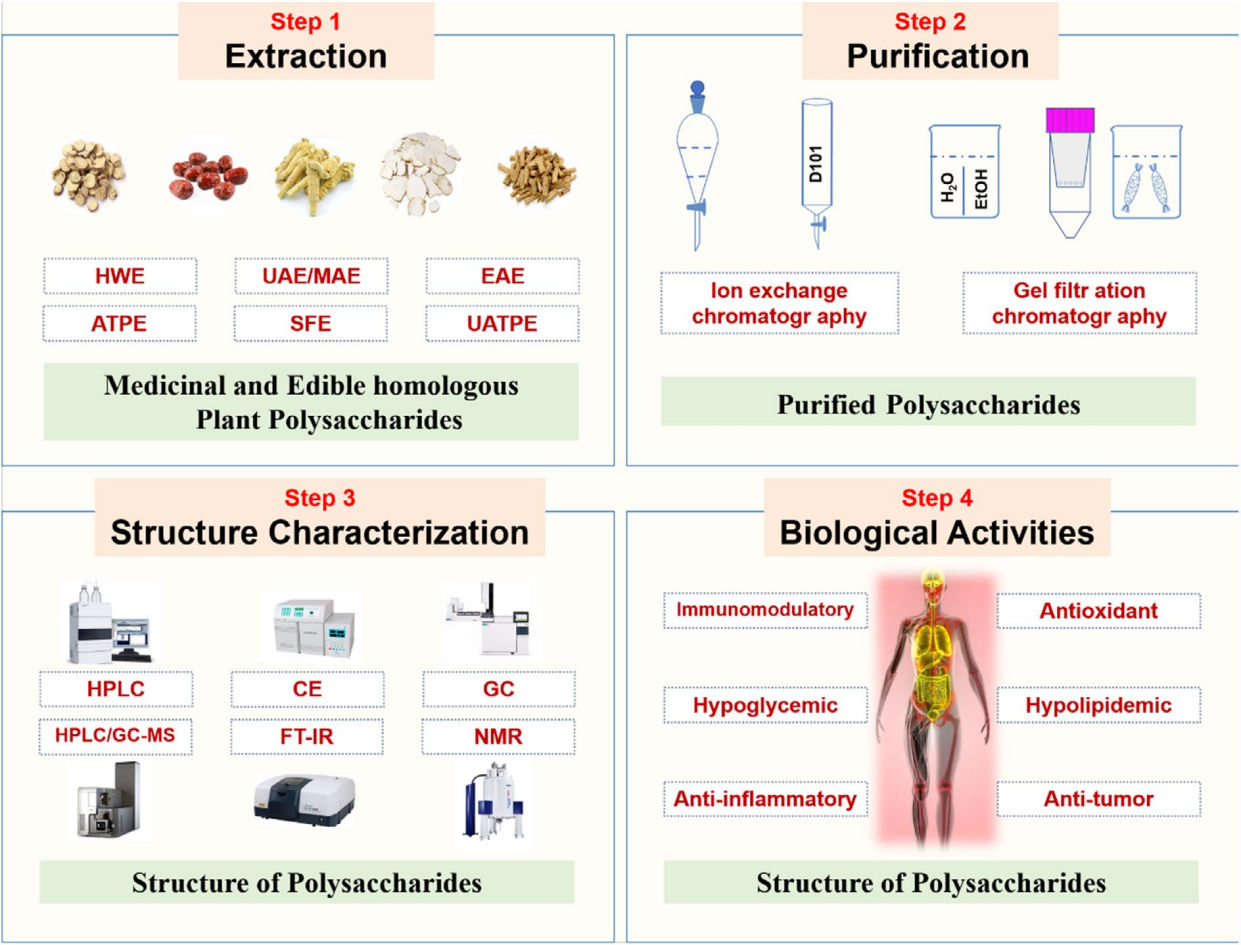
Schematic diagram of preparation, structure, and bioactivities of medicinal and edible homologous plant polysaccharides.

Due to the widespread use of MEHPPs in pharmaceuticals, health foods, and cosmetics, more and more attention has been paid to the extraction, purification, structural characterization, and biological activity of MEHPPs to improve the quality of MEHPPs products. In this paper, we systematically summarized the latest research progress of MEHPPs, expounded the principle and advantages of some methods for extraction and purification of MEHPPs, and discussed the mechanism of immune regulation, antioxidant, hypoglycemic, hypolipidemic, antitumor, and anti‐inflammatory action of MEHPPs to deeply understand the biological activity of MEHPPs (Figure 1). However, there are still gaps in understanding the structure–function relationships of MEHPPs. For example, the specific impact of minor monosaccharide components on bioactivities remains unclear, and the relationship between fine–scale structural features and polysaccharide stability during processing is yet to be fully explored. This review aims to address these gaps and provide a more comprehensive understanding.

## 2 Preparation of MEHPPs

### 2.1 Extraction of MEHPPs

After introducing the importance of MEHPPs, let's focus on their preparation methods. Extracting MEHPPs requires significant energy to break through the plant cell wall and dissolve it in the extraction solution. The traditional solvent extraction methods, such as hot water extraction (HWE), acid extraction and alkali extraction take longer extraction times and disrupt the structure of polysaccharide (Feng et al. 2020). At present, in order to overcome the disadvantages of traditional extraction methods, advanced and efficient extraction methods including ultrasonic‐assisted extraction (UAE), microwave‐assisted extraction (MAE), supercritical CO_2_ fluid extraction (SFE), enzyme‐assisted extraction (EAE), and their combination techniques have been outstanding in the extraction of MEHPPs (Wang et al. 2019). The main difference in extraction methods lies in the differences in extraction solvents and extraction instruments, each with its own advantages and disadvantages.

UAE utilizes ultrasonic waves to induce cavitation, disrupting cell walls and improving solvent penetration, offering energy efficiency but requiring optimization to prevent partial degradation, making it suitable for fibrous plant materials like Astragalus roots. MAE employs microwave energy to rapidly heat plant material, accelerating polysaccharide dissolution at high speed, though precise temperature control is needed to avoid overheating, ideal for heat‐stable polysaccharides such as those from yam. SFE uses supercritical CO_2_ as a solvent, providing high selectivity and minimal thermal damage, perfect for heat‐sensitive polysaccharides. EAE employs enzymes (e.g., cellulase, pectinase) to degrade cell walls, enhancing yield under mild conditions, but enzyme specificity must match plant composition. Combining these methods, such as UAE‐MAE or EAE‐SFE, has shown superior yields and bioactivity preservation, as evidenced by recent studies demonstrating increased polysaccharide yield from Astragalus using UAE‐MAE (Hou et al. 2020).

Hence, selecting extraction solvents and optimizing extraction time based on experimental needs and material properties is a feasible strategy to improve the extraction efficiency of MEHPPs. With the development of science and technology, the combination of multiple extraction methods is a hot topic in future polysaccharide research.

MEHs extraction solution obtained by the above method need to be filtered, concentrated, and precipitated by ethanol. In order to obtain crude MEHPPs, the water extraction–alcohol precipitation method is a feasible, convenient, and most commonly used method. The hydrogen bond between polysaccharides is broken by adding ethanol to the extraction solution, resulting in the decrease of solubility of polysaccharide in ethanol aqueous solution and precipitation. It is interesting that by adjusting the ethanol concentration, different components of crude polysaccharides can even be preliminarily purified (Yang et al. 2014). Although advanced extraction methods have shown advantages, the energy consumption and potential impact on polysaccharide structure still need to be balanced. Future research could focus on developing more energy–efficient and structure–friendly extraction techniques.

### 2.2 Purification and Isolation of MEHPPs

Now that we have covered the extraction methods, it is essential to discuss how to purify the extracted MEHPPs. The crude MEHPPs contain various impurities such as oligosaccharides, lipids, proteins, pigments, etc., which need to be further removed and purified to obtain high‐purity polysaccharides. Extract and purify crude MEHPPs by utilizing the difference in solubility of sugars in water‐ethanol, and then remove lipophilic impurities using organic reagents such as ethanol, petroleum ether, and anhydrous ether (Feng et al. 2022). Additionally, the SEVAGE (chloroform: n‐butanol volume ratio 4:1) method is commonly used for protein removal (Yang, Yang, et al. 2022). Macroporous resin D101 selectively adsorbs pigments, flavonoids, and saponins, which is more conducive to the purification of polysaccharides (Li et al. 2014). The oxidation method is based on the oxidation of H_2_O_2_ to decolorize polysaccharides (Zhang et al. 2019). The dialysis method (using an ultrafiltration filter or dialysis bag) can effectively separate polysaccharide components based on molecular weight, and this process can also achieve desalination, alcohol removal, and removal of small molecule polysaccharides and other water‐soluble impurities (Jiao et al. 2014).

Crude MEHPPs are mixtures of polysaccharide components with different molecular weights or mixtures of acidic and neutral sugars, which can be purified by stepwise precipitation (organic reagents: methanol, ethanol, and acetone; neutral salts: NaCl and (NH)_2_SO_4_), ultrafiltration, and column chromatography to obtain homogeneous polysaccharides. The most commonly used method among polysaccharide researchers is column chromatography, which has a wide range of applications in the purification of polysaccharides. Ion exchange chromatography can effectively separate and purify polysaccharides based on charge differences and is suitable for the separation of various neutral, acidic, and viscous polysaccharides. The commonly used fillers include DEAE Sepharose (Zhang, Liu, et al. 2021), DEAE Cellulose (Wang et al. 2015; Guo et al. 2023), DEAE Sephadex (Wang et al. 2013), and DEAE Sepharose Fast Flow (Wang et al. 2021; Tabarsa et al. 2022). Gel exclusion chromatography can be used to obtain homogeneous polysaccharides from crude polysaccharides by chromatographic separation according to molecular shape and size. Sepharose (Qin et al. 2018), Sephacry (Guo et al. 2023), and Sephadex (Zhang et al. 2020) are commonly used as chromatographic media to separate polysaccharides with different molecular weights.

## 3 Structural Elucidation of MEHPPs

After purification, in‐epth structural analysis of MEHPPs is crucial. Here is a detailed introduction to the key aspects of their structural elucidation. The structure characterization of the purified MHPPs homogeneous polysaccharide mainly includes four parts: (1) Monosaccharide composition (MC) (Zhao et al. 2023); (2) Molecular weight determination (MW) (Jiang et al. 2016); (3) Glycosidic bond type and position (Xie et al. 2018); (4) Spatial conformation (Xue, Li, et al. 2019). MC analysis reveals the building blocks of polysaccharides. For example, different ratios of Glc, Gal, and Ara can lead to distinct bioactivities. Molecular weight determination affects properties like solubility and viscosity. Glycosidic bond type and position determine the connectivity of monosaccharides, which is closely related to polysaccharide stability and enzyme‐ediated degradation. Spatial conformation, whether it is a linear or branched structure, also plays a vital role in biological recognition and activity. Each aspect is interrelated, and a comprehensive understanding of these relationships is essential for fully exploring the potential of MEHPPs. It was found that the structure of polysaccharides was complex a, and a variety of analytical methods were used to better understand the structure of sugars. The analytical methods used for chemical characterization of MEHP mainly include highperformance liquid chromatography (HPLC), gas chromatography (GC), capillary electrophoresis (CE), two‐dimensional nuclear magnetic resonance (^2^D‐NMR), Fourier transform infrared spectroscopy (FT‐IR), liquid chromatography–mass spectrometry (LC–MS) and gas chromatography–mass spectrometry (GC–MS) analysis.

### 3.1 Monosaccharide Composition

It is worth noting that the study of MC plays a crucial role in the structural identification of polysaccharides. Firstly, complete or partial acid hydrolysis is performed on MEHPP to break down glycosidic bonds. After neutralization, filtration, and derivatization, products can be generated for instrument detection. Nowadays, GC (Yang, Zhai, et al. 2022) and HPLC (Ma et al. 2017) are the most commonly used MC analysis methods. To sum up, current knowledge with respect to the monosaccharides in MEHPPs involves Glucose (Glc), fructose (Fru) fucose (Fuc), Galactose (Gal), Rhamnose (Rha), Mannose (Mannose) (Man), Arabinose (Ara), Xylose (Xyl), and Glucuronic acid (GlcA) (Wu et al. 2022). For instance, a study by Smith et al. (Malinowska et al. 2010). on Astragalus polysaccharides further confirmed the presence of Glc, Ara, and Gal, and also found trace amounts of Man in some samples, expanding our understanding of monosaccharide diversity in MEHPPs.

### 3.2 Molecular Weight

Notably, the chemical properties of MEHPPs are closely related to their average molecular weight (Jiang et al. 2016). Highperformance gel permeation chromatography (HPGPC) (He et al. 2023), highperformance size exclusion chromatography (HPSEC) (Chen, Zhu, Chen, et al. 2022), and highperformance gel filtration chromatography (Yu et al. 2017) are usually used for polysaccharides analysis and provide molar mass distribution of polysaccharides. In particular, chromatographic separation and the combination of multiple detectors demonstrate powerful functionality, providing various parameters such as MW, polydispersity index (MW/MN) simultaneously. Currently, refractive index detector (RID) (Chen, Zhu, Peng, et al. 2022), ELSD (Shao et al. 2024), MALLS (Zhou et al. 2020) and differential pressure viscosity‐angle laser light scattering detector (Ciric et al. 2012) have been widely used for molecular weight calibration. According to Malinowska et al. (2010). HPGPC combined with a multi‐ngle laser light scattering detector can more accurately measure the molecular weight distribution of complex polysaccharide mixtures.

### 3.3 Glycosidic Bond Type and Position

At present, three main methods to determine the location of the glycosidic bond of the MEHPP structure, the degree of polymerization with the number of branch chains of linear polysaccharide are ^2^D‐NMR (Zhou et al. 2021), GC–MS (Yang et al. 2023), LC–MS (Li et al. 2021) and FT‐IR (Zou et al. 2022). Methylation analysis (Nagar et al. 2020), periodate oxidation (Pandeirada et al. 2022) and Smith degradation (Cheng et al. 2023) are important chemical methods to study the structure of sugars, which are widely used to determine the linkage position of monosaccharide residues in oligosaccharides and polysaccharides. Moreover, methylation reaction, GC–MS, coupled with ^2^D‐NMR spectroscopy provides a feasible solution to analyze the linking sequence, linking sites, and substitutional functional groups of sugars, and to infer the main chain, branch chain, and repeating units of polysaccharides.

The integration of two‐dimensional nuclear magnetic resonance (2D‐NMR) and mass spectrometry (MS) technologies provides a multiscale analytical approach for resolving the fine structural details of medicinal and edible homologous plant polysaccharides (MEHPPs). In NMR analysis, COSY and TOCSY spectra elucidate vicinal coupling relationships between monosaccharide residues (Bai et al. 2020), whereas HSQC distinguishes α/β glycosidic bond configurations via carbon‐hydrogen correlations. HMBC further identifies glycosidic linkage positions through long‐range coupling (2–3 J), particularly effective for resolving stereochemical features of complex branching structures and repeating units (Wu and McClements 2015). Complementing these findings, MS techniques deliver molecular weight and sequence information: MALDI‐TOF MS employs soft ionization to minimize fragmentation, enabling precise determination of high‐molecular‐weight polysaccharide distributions. LC/GC–MS coupled with trifluoroacetic acid (TFA) hydrolysis and 1‐phenyl‐3‐methyl‐5‐pyrazolone (PMP) derivatization achieves pmol‐level sensitivity in monosaccharide compositional analysis. Tandem MS (MS/MS) generates diagnostic fragment ions via collision‐induced dissociation (CID), allowing deduction of glycosidic bond positions and sequences—for example, β(1 → 3) galactose linkages can be identified through B‐ion series (characterized by CH₂O₅ group loss).

In practice, researchers typically implement a four‐stage validation workflow (Zhou, Yin, et al. 2023): Complete TFA hydrolysis followed by PMP derivatization and LC–MS analysis for monosaccharide profiling. Permethylation and GC–MS analysis to map glycosidic linkage positions. Cross‐validation using 2D‐NMR data to confirm hydroxyl substitution patterns, anomeric carbon configurations, and spatial arrangements.

### 3.4 Sugar Ring and Anomeric Carbon Configuration

The determination of sugar ring and telomere carbon configuration is to clarify the relationship between monosaccharides that constitute polysaccharides. FT‐IR is commonly used for simultaneous determination of α‐ or β‐ Type glycosidic bonds (Shu et al. 2018), furan or pyran type sugars (Wang et al. 2012). In addition, the development of advanced structural research methods such as GC–MS, CE–MS (Deng et al. 2023), MALDI‐TOF MS, and ^2^D‐NMR contributes to a comprehensive and accurate understanding of the primary structure of polysaccharides.

## 4 Biological Activities of MEHPPs

Polysaccharides, as one of the important active ingredients of MEH traditional Chinese medicine, have various effects such as immune regulation, antioxidant, hypoglycemic, lipid‐lowering, antitumor, anti‐inflammatory, etc. Natural MEHPP has low toxicity and high safety, and is increasingly used in pharmaceutical products, health food, and cosmetics. In recent years, many researchers have focused on exploring and developing chronic disease prevention and treatment drugs with unique efficacy and low toxicity side effects from MEHPPs.

### 4.1 Immunomodulatory Activity

The immunomodulatory activity of MEHPPs is considered to be an important tool to resist the invasion of cancer and improve the body's resistance to disease. Nowadays, polysaccharides mainly exert their immunomodulatory activity by promoting the proliferation and differentiation of immune cells, inducing the release of cytokines, and regulating the structure of gut microbiota. Hu et al. summarized the immunomodulatory activity of ginseng polysaccharide, which promoted the proliferation of dendritic cells (DC), stimulated T lymphocytes, and initiated immune response. Furthermore, ginseng polysaccharide significantly enhanced canavalin A‐ and lipopolysaccharide‐induced T cell and B cell proliferation, respectively, thereby enhancing cellular and humoral immunity (Hu et al. 2022). He et al. (2016) found that Dendrobium polysaccharide stimulated phagocytic cells to release cytokines (TNF‐α, IL‐1β, IL‐10), induced the immune activity of ERK1/2 and NF‐κB, and thus showed significant immune‐enhancing properties. Li et al. found that *Dendrobium dendrobium* polysaccharide improved the microbial community diversity in the colon of immunosuppressed mice and normal mice, playing an immunomodulatory role. *Dendrobium dendrobium* polysaccharide modulates immunosuppression both in immunosuppressed mice and normal mice by improving microbial community diversity and restoring disturbed gut microbial community structure (Li et al. 2024). Commonly, MEHPPs activate NF‐κB pathways to boost cytokine production, but differences arise from structural variations, with ginseng's high Glc content enhancing T‐cell proliferation more than Dendrobium's Ara‐rich polysaccharides, which favor microbiota modulation.

### 4.2 Antioxidant Activity

As is well known, reactive oxygen species (ROS) are involved in the mechanism of killing viruses and bacteria in the body. However, excess ROS triggers oxidative stress; oxidative damage occurs to DNA, RNA, lipids, and proteins (Fleming and Luo 2021). The oxidative stress has been linked to many adverse health problems, such as neurodegenerative disease, cardiovascular disease, allergies, and aging. Therefore, dietary antioxidant supplementation is necessary to reduce the risk of related diseases. MEHPPs are considered one of the potential natural sources of antioxidants, which have a protective effect on oxidative stress by clearing oxidative free radicals and activating various antioxidant enzymes. Zhou et al. extracted crude polysaccharides from yam (YPs) by HWE method and purified the YPs using anion exchange chromatography (DEAE‐cellulose column chromatography) to obtain a single polysaccharide component. The antioxidant activity of YPs was evaluated based on its scavenging effect on hydroxyl radical. The results showed that the polysaccharide had *α*‐*D*‐Gluc‐(1 → 4) glycosidic bond, and the *C2* hydroxyl group was replaced by ethoxy. Additionally, these findings provide a theoretical basis and practical support for the application of yam polysaccharide as a functional antioxidant food (Malinowska et al. 2010). Furthermore, some researchers extracted polysaccharides from *Astragalus*, *Panax ginseng* , *Glycyrrhiza*, and *Lycium chinensis* to improve antioxidant activity by increasing antioxidant oxidase activity (glutathione peroxidase, GSH‐Px; total superoxide dismutase, T‐SOD, and total antioxidant capacity, T‐AOC) (Fleming and Luo 2021; Zhao et al. 2024; Simayi et al. 2021). Across plant sources, MEHPPs upregulate GSH‐Px and T‐SOD, but Lycium polysaccharides excel in ROS scavenging due to their high GlcA content, unlike Astragalus's Gal‐rich structures, which prioritize enzyme activation.

### 4.3 Hypoglycemic Activity

Nowadays, diabetes is mainly characterized by elevated blood sugar concentration, which has become a worldwide public health problem. Diabetic patients are prone to diabetic cardiovascular disease, diabetic nephropathy, etc., seriously endangers human health. It is worth note that MEH is edible and safe and suitable for use as a food, health supplement or medicine to treat and prevent diabetes. On the one hand, MEHPPs have the ability to inhibite *α‐amylase* and *α‐glucosidase* activities and decrease postprandial blood glucose; on the other hand, MEHPPs enhance insulin sensitivity, regulating insulin signaling and improving insulin resistance via downregulating PTP1B expression and regulating PI3K‐AKT/GSK3β/FoxO1 signaling pathway. Ren et al. confirmed that *Mulberry* leaf polysaccharide (MLPII) insulin signaling by inhibiting the expression of PTP1B, activating the PI3K‐AKT pathway and alleviate oxidative stress in the livers of rats with with Type 2 diabetes mellitus (Xue, Hu, et al. 2019). Luo et al. evaluated the therapeutic effect of *Pueraria lobata* root polysaccharide (PLP) on diabetic metabolic syndrome, and found that PLP down‐regulates the mRNA expression of PEPCK, G6PC, FOXO1, SREBP‐1, and ACC in liver, which plays an antidiabetic role via activating PI3K/AKT signaling pathway, thereby improving insulin resistance, glucose and lipid metabolism in db/db mice (Ren et al. 2015). Most MEHPPs target PI3K‐AKT pathways, but Mulberry's high Rha content enhances α‐glucosidase inhibition compared to Pueraria's Glc‐dominated structures, which focus on insulin signaling.

### 4.4 Antitumor Activity

Surgery, chemotherapy, and radiotherapy are effective measures for most cancers, but side effects including low immunity and organ failure lead to a serious reduction in patients' quality of life, which is difficult to solve in clinical medicine. Growing studies have shown that MEHPPs inhibit the occurrence and development of tumors through multi‐target and multi‐pathway mechanisms, and have anticancer activity both in vivo and in vitro. The antitumor mechanism of MEHPPs is mainly divided into two kinds: (1) MEHPPs directly act on tumor cells, change the expression levels of key proteins and enzymes in tumor cell signal transduction pathways, inhibit tumor cell growth, proliferation, migration, and invasion to induce tumor cell apoptosis; (2) MEHPPs, as a biological immune response modulators, enhance the immune function of the body by promoting the activity of LAK and natural killer cells (NK), inducing macrophages to produce tumor necrosis factor, and indirectly inhibit or kill tumor cells. Zhao et al. isolated a new alkali‐soluble polysaccharide (AASP) from *Angelica sinensis*, and the primary structure of the polysaccharide was characterized by Smith degradation, methylation, FT‐IR, and NMR analysis. In vitro antitumor activity experiments showed that AASP exhibited a significant antitumor effect on H22 hepatocellular carcinoma cells, with an inhibition rate of 48.57%. Besides, AASP also stimulated the proliferation of immune cells (splenocytes, peritoneal macrophages and natural killer cells), and assisted cytokine release (TNF‐α, IL‐2, and IFN‐γ) to induce apoptosis of H22 solid tumor cells during the G0/G1 phase (Luo et al. 2021). Zhao et al. prepared a monodisperse and stable spherical nanoparticle (Tw‐TMP‐SeNP, 50 nm) using inulin frutin (TMP50‐2) from dandelion, which can significantly inhibit the proliferation of cancer cells (HepG2, A549, and HeLa) in vitro. In addition, Tw‐TMP‐SeNP can restrain tumor proliferation and migration in zebrafish xenografts and inhibit neovascularization of transgenic zebrafish (Zhao et al. 2021). MEHPPs commonly upregulate TNF‐α and IL‐2, but Angelica's β‐pyranose structures directly induce apoptosis, whereas Dandelion's nanoparticle forms excel in migration inhibition. In the field of oncology, MEHPPs enable precise integration with nanomaterials through their molecular self‐assembly properties. For instance, as biocompatible carriers for polyoxometalate‐nanozyme integrated nanomotors (POMotors), their immunomodulatory properties enhance tumor microenvironment targeting while reducing systemic toxicity, achieving precision therapy through photothermal‐catalytic synergy (Zhang, Song, et al. 2021). When combined with tungsten carbide‐loaded macrophages, MEHPPs not only activate macrophages to improve tumor penetration but also significantly amplify photothermal conversion efficiency. Animal studies confirm over 40% enhancement in antitumor efficacy (Tang et al. 2024). At the mechanistic level, MEHPPs exhibit dual regulatory capabilities when integrated with metal‐phenolic networks. Their phenolic hydroxyl domains induce ferroptosis in colorectal cancer cells, whereas released immunomodulatory molecules activate systemic immune responses through TLR4/NF‐κB pathways, establishing a therapeutic‐immunological positive feedback loop (Gao et al. 2019). Notably, MEHPPs demonstrate structural adaptability in nanozyme engineering applications. When complexed with Clusterphene nanozymes, their phenolic networks stabilize enzymatic active sites and prolong circulation time, achieving a 2.3‐fold enhancement in reactive oxygen species (ROS) generation efficiency, thereby creating a novel catalytic platform for targeted therapy (Li et al. 2025).

### 4.5 Anti‐Inflammatory Activity

It is generally recognized that inflammation is a highly coordinated response of the body to microbial infection, tissue damage, toxins and other stimulating factors. Moderate inflammation is beneficial to the body, otherwise excessive inflammation will lead to atherosclerosis and hypertension and other diseases. The modern researches indicated that MEHPPs exert anti‐inflammatory effects by inhibiting the expression of related target genes in multiple signaling pathways (NF‐κB/MAPK/STAT3) to reduce the release of inflammatory mediators. Jia et al. found that an alkali‐extracted polysaccharide from *Dioscorea opposita* Thunb (HY‐B) could inhibit the production of NO and TNF‐α in RAW 264.7 macrophage stimulated by lipopolysaccharides (LPSs) (Yue et al. 2024). Zhou et al. investigated the anti‐inflammatory mechanism of dandelion polysaccharide is to reduce the expression of chemokine *Mcp‐1* and pro‐inflammatory cytokines (TNF‐α, Il‐1β and Il‐6). In addition, dandelion polysaccharides may play an important role in the reduction of atherosclerosis through its antioxidant and anti‐inflammatory properties (Jia et al. 2021). NF‐κB inhibition is a shared mechanism, but Dioscorea's high Man content targets macrophage activation, whereas Dandelion's GlcA‐rich structures emphasize cytokine suppression. Recent studies highlight novel anti‐inflammatory MEHPPs from Codonopsis with enhanced STAT3 inhibition (Zhou, Wang, et al. 2023).

## 5 Conclusions

In recent years, MEHPPs have attracted much attention and research interest from biologists because of their immunomodulatory, antioxidant, hypoglycemic, hypolipidemic, antitumor, and anti‐inflammatory activities. In order to effectively extract plant polysaccharides from MEHP, many researchers draw on modern advanced science and technology such as UAE, MAE, SFE, EAE, and their combination technology to obtain crude MEHPPs. Of course, the traditional UAE and MAE methods have low requirements for instruments and equipment and still play an important role in the process of polysaccharide extraction from MEHP. The achieved crude polysaccharide needs to be further removed from the fat, protein pigments, etc., to obtain high‐purity polysaccharide. Despite significant advances in the separation and purification technology of MEHP, there are still many opportunities and challenges that need to be addressed. At present, the structural analysis of MEHP mainly focuses on MC, molecular weight, monosaccharide sequence, type of glycosidic bond, and location of glycosidic bond, etc., and there is a lack of technical means to characterize the dynamic changes of its advanced structure.

Innovative extraction methods, such as pulsed—electric field extraction and ionic liquid—based extraction, show promise for improving extraction efficiency and polysaccharide quality. Pulsed—electric field extraction can disrupt cell walls with less heat—induced degradation, and ionic liquids can selectively extract specific polysaccharides. Regarding potential applications, MEHPPs could be further explored in tissue engineering, where their biocompatibility and bioactivity may contribute to the development of novel scaffolds for cell growth and tissue regeneration.

Compared to common polysaccharides like starch or cellulose, MEHPPs exhibit unique structural features, such as specific glycosidic linkages (e.g., α‐D‐Glc‐(1 → 4) in yam) and higher branching, which enhance bioactivities like immunomodulation and antioxidant effects. For instance, ginseng polysaccharides with Glc‐Gal compositions outperform cellulose's Glc‐dominated structures in immune stimulation, making MEHPPs ideal for functional foods and therapeutics due to their dual medicinal‐edible nature. Future research should explore innovative extraction combinations (e.g., UAE‐EAE) and novel applications, such as targeted drug delivery or gut microbiota modulation, leveraging recent advancements in structural analysis (Ji et al. 2020).

## Author Contributions

**Mengnan Chen:** conceptualization (equal), data curation (equal), methodology (equal), resources (equal), software (equal), writing – original draft (equal), writing – review and editing (equal). **Wenfeng Wei:** formal analysis (equal), funding acquisition (equal), supervision (equal), visualization (equal). **Zhuang Wang:** data curation (equal), methodology (equal), visualization (equal). **Weiming Wang:** funding acquisition (equal), project administration (equal), resources (equal).

## Conflicts of Interest

The authors declare no conflicts of interest.

## Data Availability

The authors have nothing to report.

## References

[fsn370453-bib-0001] Bai, Y. , X. Jia , F. Huang , et al. 2020. “Structural Elucidation, Anti‐Inflammatory Activity and Intestinal Barrier Protection of Longan Pulp Polysaccharide LPIIa.” Carbohydrate Polymers 246: 116532. 10.1016/j.carbpol.2020.116532. 32747231

[fsn370453-bib-0003] Chen, Z. , B. Zhu , Z. Chen , et al. 2022. “Effects of Steam on Polysaccharides From Polygonatum Cyrtonema Based on Saccharide Mapping Analysis and Pharmacological Activity Assays.” Chinese Medicine 17, no. 1: 97. 10.1186/s13020-022-00650-3. 35978410 PMC9386940

[fsn370453-bib-0004] Chen, Z. , B. Zhu , X. Peng , S. Li , and J. Zhao . 2022. “Quality Evaluation of *Ophiopogon japonicus* From Two Authentic Geographical Origins in China Based on Physicochemical and Pharmacological Properties of Their Polysaccharides.” Biomolecules 12, no. 10: 1491. 10.3390/biom12101491. 36291700 PMC9599291

[fsn370453-bib-0005] Cheng, N. , H. Wang , H. Hao , F.‐U. Rahman , and Y. Zhang . 2023. “Research Progress on Polysaccharide Components of *Cistanche Deserticola* as Potential Pharmaceutical Agents.” European Journal of Medicinal Chemistry 245: 114892. 10.1016/j.ejmech.2022.114892. 36334326

[fsn370453-bib-0006] Cheng, Q. , S. Peng , F. Li , et al. 2021. “Quality Distinguish of Red Ginseng From Different Origins by HPLC–ELSD/PDA Combined With HPSEC–MALLS–RID, Focus on the Sugar‐Markers.” Separations 8, no. 11: 198. 10.3390/separations8110198.

[fsn370453-bib-0007] Cheong, K.‐L. , D.‐T. Wu , J. Zhao , and S.‐P. Li . 2015. “A Rapid and Accurate Method for the Quantitative Estimation of Natural Polysaccharides and Their Fractions Using High Performance Size Exclusion Chromatography Coupled With Multi‐Angle Laser Light Scattering and Refractive Index Detector.” Journal of Chromatography A 1400: 98–106. 10.1016/j.chroma.2015.04.054. 25990349

[fsn370453-bib-0008] Ciric, J. , J. Oostland , J. W. De Vries , A. J. J. Woortman , and K. Loos . 2012. “Size Exclusion Chromatography With Multi Detection in Combination With Matrix‐Assisted Laser Desorption Ionization‐Time‐Of‐Flight Mass Spectrometry as a Tool for Unraveling the Mechanism of the Enzymatic Polymerization of Polysaccharides.” Analytical Chemistry 84, no. 23: 10463–10470. 10.1021/ac302704q. 23121513

[fsn370453-bib-0009] Corrado, A. , M. De Martino , V. Bordoni , et al. 2023. “A Universal UHPLC‐CAD Platform for the Quantification of Polysaccharide Antigens.” Scientific Reports 13, no. 1: 10646. 10.1038/s41598-023-37832-4. 37391501 PMC10313704

[fsn370453-bib-0010] Deng, Y. , J. Zhao , and S. Li . 2023. “Quantitative Estimation of Enzymatic Released Specific Oligosaccharides From Hericium Erinaceus Polysaccharides Using CE‐LIF.” Journal of Pharmaceutical Analysis 13, no. 2: 201–208. 10.1016/j.jpha.2022.11.004. 36908854 PMC9999295

[fsn370453-bib-0011] Feng, J.‐Y. , Y.‐Q. Xie , P. Zhang , et al. 2022. “Hepatoprotective Polysaccharides From *Geranium wilfordii*: Purification, Structural Characterization, and Their Mechanism.” Molecules 27, no. 11: 3602. 10.3390/molecules27113602. 35684541 PMC9182495

[fsn370453-bib-0012] Feng, Y. , J. Zhang , C. Wen , et al. 2020. “Recent Advances in Agaricus Bisporus Polysaccharides: Extraction, Purification, Physicochemical Characterization and Bioactivities.” Process Biochemistry 94: 39–50. 10.1016/j.procbio.2020.04.010.

[fsn370453-bib-0013] Fleming, E. , and Y. Luo . 2021. “Co‐Delivery of Synergistic Antioxidants From Food Sources for the Prevention of Oxidative Stress.” Journal of Agriculture and Food Research 3: 100107. 10.1016/j.jafr.2021.100107.

[fsn370453-bib-0014] Gao, Y. , W. Huang , C. Yang , et al. 2019. “Targeted Photothermal Therapy of Mice and Rabbits Realized by Macrophage‐Loaded Tungsten Carbide.” Biomaterials Science 7, no. 12: 5350–5358. 10.1039/c9bm00911f. 31620706

[fsn370453-bib-0015] Guo, D. , X. Yin , D. Wu , J. Chen , and X. Ye . 2023. “Natural Polysaccharides From Glycyrrhiza Uralensis Residues With Typical Glucan Structure Showing Inhibition on α‐Glucosidase Activities.” International Journal of Biological Macromolecules 224: 776–785. 10.1016/j.ijbiomac.2022.10.165. 36280177

[fsn370453-bib-0016] He, T.‐B. , Y.‐P. Huang , L. Yang , et al. 2016. “Structural Characterization and Immunomodulating Activity of Polysaccharide From Dendrobium Officinale.” International Journal of Biological Macromolecules 83: 34–41. 10.1016/j.ijbiomac.2015.11.038. 26592697

[fsn370453-bib-0017] He, Y. , S. Huang , G. Xu , et al. 2023. “Structural Characteristics and Immunomodulation Activity of a Polysaccharide From Purslane ( *Portulaca Oleracea* ).” Journal of Functional Foods 109: 105781. 10.1016/j.jff.2023.105781.

[fsn370453-bib-0018] Hou, C. , L. Chen , L. Yang , and X. Ji . 2020. “An Insight Into Anti‐Inflammatory Effects of Natural Polysaccharides.” International Journal of Biological Macromolecules 153: 248–255. 10.1016/j.ijbiomac.2020.02.315. 32114173

[fsn370453-bib-0019] Hu, Y. , Y. He , Z. Niu , et al. 2022. “A Review of the Immunomodulatory Activities of Polysaccharides Isolated From Panax Species.” Journal of Ginseng Research 46, no. 1: 23–32. 10.1016/j.jgr.2021.06.003. 35058724 PMC8753523

[fsn370453-bib-0020] Ji, X. , M. Yin , H. Nie , and Y. Liu . 2020. “A Review of Isolation, Chemical Properties, and Bioactivities of Polysaccharides From *Bletilla striata* .” BioMed Research International 2020: 5391379. 10.1155/2020/5391379. 32596325 PMC7273373

[fsn370453-bib-0021] Jia, X. , X. Wang , Y. Liu , et al. 2021. “Structural Characterization of an Alkali‐Extracted Polysaccharide From *Dioscorea opposita* Thunb. With Initial Studies on Its Anti‐Inflammatory Activity.” Journal of Carbohydrate Chemistry 40, no. 6: 308–324.

[fsn370453-bib-0022] Jiang, Y. , X. Qi , K. Gao , et al. 2016. “Relationship Between Molecular Weight, Monosaccharide Composition and Immunobiologic Activity of Astragalus Polysaccharides.” Glycoconjugate Journal 33: 755–761. 10.1007/s10719-016-9669-z. 27129881

[fsn370453-bib-0023] Jiao, L. , B. Li , M. Wang , Z. Liu , X. Zhang , and S. Liu . 2014. “Antioxidant Activities of the Oligosaccharides From the Roots, Flowers and Leaves of *Panax ginseng* C.A. Meyer.” Carbohydrate Polymers 106: 293–298. 10.1016/j.carbpol.2014.02.035. 24721081

[fsn370453-bib-0024] Li, C. L. , X. L. Shi , Y. Men , L. Yang , and A. M. Yang . 2014. “Purification of Astragalus Polysaccharide With Macroporous Resins.” Applied Mechanics and Materials 618: 326–329. 10.4028/www.scientific.net/AMM.618.326.

[fsn370453-bib-0025] Li, J. , W. Tao , W. Zhou , et al. 2024. “Dendrobium Officinale Leaf Polysaccharide Has a Dual Effect of Alleviating the Syndromes of Immunosuppressed Mice and Modulating Immune System of Normal Mice.” Journal of Functional Foods 113: 105974. 10.1016/j.jff.2023.105974.

[fsn370453-bib-0026] Li, X. , J. Liu , T. Zuo , et al. 2022. “Advances and Challenges in Ginseng Research From 2011 to 2020: The Phytochemistry, Quality Control, Metabolism, and Biosynthesis.” Natural Product Reports 39, no. 4: 875–909. 10.1039/D1NP00071C. 35128553

[fsn370453-bib-0027] Li, Y. , Y. Duan , Y. Li , et al. 2025. “Cascade Loop of Ferroptosis Induction and Immunotherapy Based on Metal‐Phenolic Networks for Combined Therapy of Colorectal Cancer.” Exploration 5, no. 1: 20230117. 10.1002/exp.20230117. 40040829 PMC11875444

[fsn370453-bib-0028] Li, Y. , J. Liang , J.‐N. Gao , Y. Shen , H.‐X. Kuang , and Y.‐G. Xia . 2021. “A Novel LC‐MS/MS Method for Complete Composition Analysis of Polysaccharides by Aldononitrile Acetate and Multiple Reaction Monitoring.” Carbohydrate Polymers 272: 118478. 10.1016/j.carbpol.2021.118478. 34420737

[fsn370453-bib-0029] Liu, J. , H. Wang , F. Yang , et al. 2022. “Multi‐Level Fingerprinting and Cardiomyocyte Protection Evaluation for Comparing Polysaccharides From Six Panax Herbal Medicines.” Carbohydrate Polymers 277: 118867. 10.1016/j.carbpol.2021.118867. 34893272

[fsn370453-bib-0030] Luo, D. , X. Dong , J. Huang , C. Huang , G. Fang , and Y. Huang . 2021. “ *Pueraria lobata* Root Polysaccharide Alleviates Glucose and Lipid Metabolic Dysfunction in Diabetic Db/Db Mice.” Pharmaceutical Biology 59, no. 1: 380–388. 10.1080/13880209.2021.1898648. PMC8018507 33794128

[fsn370453-bib-0031] Ma, X.‐L. , F. F. Song , H. Zhang , X. Huan , and S. Y. Li . 2017. “Compositional Monosaccharide Analysis of *Morus nigra* Linn by HPLC and HPCE Quantitative Determination and Comparison of Polysaccharide From *Morus nigra* Linn by HPCE and HPLC.” Current Pharmaceutical Analysis 13, no. 5: 433–437. 10.2174/1573412913666170330150807. 29213223 PMC5684802

[fsn370453-bib-0032] Malinowska, E. , W. Krzyczkowski , G. Łapienis , and F. Herold . 2010. “Densitometric Determination of Carbohydrates: Application to Purification and Molecular Weight Determination of Polysaccharide From Hericium Erinaceum Mushroom.” Food Research International 43, no. 4: 988–995. 10.1016/j.foodres.2010.01.011.

[fsn370453-bib-0033] Nagar, S. , A. K. Lakhera , and V. Kumar . 2020. “Upgrading Methylation Method for Structural Studies of Polysaccharides: Case Analysis of a Bioactive Polysaccharide From *Acacia tortilis* .” Journal of Biologically Active Products From Nature 10, no. 2: 70–85. 10.1080/22311866.2020.1767688.

[fsn370453-bib-0034] Pandeirada, C. O. , J. A. Hageman , H.‐G. Janssen , Y. Westphal , and H. A. Schols . 2022. “Identification of Plant Polysaccharides by MALDI‐TOF MS Fingerprinting After Periodate Oxidation and Thermal Hydrolysis.” Carbohydrate Polymers 292: 119685. 10.1016/j.carbpol.2022.119685. 35725177

[fsn370453-bib-0035] Qin, X. , X. Fan , L. Zhang , H. Zheng , C. Zhang , and J. Yuan . 2018. “Extraction, Purification, and Structure Characterization of Polysaccharides From Crassostrea Rivularis.” Food Science & Nutrition 6, no. 6: 1621–1628. 10.1002/fsn3.695. 30258605 PMC6145277

[fsn370453-bib-0036] Ren, C. , Y. Zhang , W. Cui , et al. 2015. “A Polysaccharide Extract of Mulberry Leaf Ameliorates Hepatic Glucose Metabolism and Insulin Signaling in Rats With Type 2 Diabetes Induced by High Fat‐Diet and Streptozotocin.” International Journal of Biological Macromolecules 72: 951–959. 10.1016/j.ijbiomac.2014.09.060. 25316427

[fsn370453-bib-0037] Shao, S. , X. Si , Y. Zhang , J. Li , P. Tu , and Q. Zhang . 2024. “Multiple Fingerprint and Pattern Recognition Analysis on Polysaccharides of Four Edible Mushrooms.” International Journal of Biological Macromolecules 259: 129236. 10.1016/j.ijbiomac.2024.129236. 38184032

[fsn370453-bib-0038] Shu, G. , S. Jiang , J. Mu , H. Yu , H. Duan , and X. Deng . 2018. “Antitumor Immunostimulatory Activity of Polysaccharides From *Panax japonicus* C. A. Mey: Roles of Their Effects on CD4 + T Cells and Tumor Associated Macrophages.” International Journal of Biological Macromolecules 111: 430–439. 10.1016/j.ijbiomac.2018.01.011. 29317237

[fsn370453-bib-0039] Simayi, Z. , P. Rozi , X. Yang , et al. 2021. “Isolation, Structural Characterization, Biological Activity, and Application of Glycyrrhiza Polysaccharides: Systematic Review.” International Journal of Biological Macromolecules 183: 387–398. 10.1016/j.ijbiomac.2021.04.099. 33887291

[fsn370453-bib-0040] Tabarsa, M. , A. Jafari , S. You , and R. Cao . 2022. “Immunostimulatory Effects of a Polysaccharide From *Pimpinella anisum* Seeds on RAW264.7 and NK‐92 Cells.” International Journal of Biological Macromolecules 213: 546–554. 10.1016/j.ijbiomac.2022.05.174. 35660044

[fsn370453-bib-0041] Tang, M. , J. Ni , Z. Yue , et al. 2024. “Polyoxometalate‐Nanozyme‐Integrated Nanomotors (POMotors) for Self‐Propulsion‐Promoted Synergistic Photothermal‐Catalytic Tumor Therapy.” Angewandte Chemie International Edition in English 63, no. 6: e202315031. 10.1002/anie.202315031. 38117015

[fsn370453-bib-0042] Wang, H. , X. Wang , L. Zhou , et al. 2021. “Structural Characteristics and In Vitro and In Vivo Immunoregulatory Properties of a Gluco‐Arabinan From Angelica Dahurica.” International Journal of Biological Macromolecules 183: 90–100. 10.1016/j.ijbiomac.2021.04.077. 33872613

[fsn370453-bib-0043] Wang, R. , P. Chen , F. Jia , J. Tang , F. Ma , and B. Xu . 2012. “Characterization and Antioxidant Activities of Polysaccharides From *Panax japonicus* C.A. Meyer.” Carbohydrate Polymers 88, no. 4: 1402–1406. 10.1016/j.carbpol.2012.02.026.

[fsn370453-bib-0044] Wang, X.‐Y. , D. Zhang , J.‐Y. Yin , S.‐P. Nie , and M.‐Y. Xie . 2019. “Recent Developments in Hericium Erinaceus Polysaccharides: Extraction, Purification, Structural Characteristics and Biological Activities.” Critical Reviews in Food Science and Nutrition 59, no. sup1: S96–S115. 10.1080/10408398.2018.1521370. 30421988

[fsn370453-bib-0045] Wang, Y. , Y. Chen , H. Xu , H. Luo , and R. Jiang . 2013. “Analgesic Effects of Glycoproteins From *Panax ginseng* Root in Mice.” Journal of Ethnopharmacology 148, no. 3: 946–950. 10.1016/j.jep.2013.05.049. 23747537

[fsn370453-bib-0046] Wang, Y. , M. Huang , R. Sun , and L. Pan . 2015. “Extraction, Characterization of a Ginseng Fruits Polysaccharide and Its Immune Modulating Activities in Rats With Lewis Lung Carcinoma.” Carbohydrate Polymers 127: 215–221. 10.1016/j.carbpol.2015.03.070. 25965477

[fsn370453-bib-0047] Wu, B.‐C. , and D. J. McClements . 2015. “Microgels Formed by Electrostatic Complexation of Gelatin and OSA Starch: Potential Fat or Starch Mimetics.” Food Hydrocolloids 47: 87–93. 10.1016/j.foodhyd.2015.01.021.

[fsn370453-bib-0048] Wu, Y. , H. Liu , Z. Li , et al. 2022. “Purification of Polysaccharides From *Phellinus linteus* by Using an Aqueous Two‐Phase System and Evaluation of the Physicochemical and Antioxidant Properties of Polysaccharides In Vitro.” Preparative Biochemistry & Biotechnology 52, no. 1: 89–98. 10.1080/10826068.2021.1911815. 33939578

[fsn370453-bib-0049] Xie, S.‐Z. , J.‐C. Ge , F. Li , et al. 2018. “Digestive Behavior of Dendrobium Huoshanense Polysaccharides in the Gastrointestinal Tracts of Mice.” International Journal of Biological Macromolecules 107: 825–832. 10.1016/j.ijbiomac.2017.09.047. 28923569

[fsn370453-bib-0050] Xu, J. , J. Zhang , Y. Sang , et al. 2022. “Polysaccharides From Medicine and Food Homology Materials: A Review on Their Extraction, Purification, Structure, and Biological Activities.” Molecules 27, no. 10: 3215. 10.3390/molecules27103215. 35630690 PMC9147777

[fsn370453-bib-0051] Xue, H. , P. Li , J. Bian , Y. Gao , Y. Sang , and J. Tan . 2022. “Extraction, Purification, Structure, Modification, and Biological Activity of Traditional Chinese Medicine Polysaccharides: A Review.” Frontiers in Nutrition 9: 1005181. 10.3389/fnut.2022.1005181. 36159471 PMC9505017

[fsn370453-bib-0052] Xue, H. , W. Wang , J. Bian , Y. Gao , Z. Hao , and J. Tan . 2022. “Recent Advances in Medicinal and Edible Homologous Polysaccharides: Extraction, Purification, Structure, Modification, and Biological Activities.” International Journal of Biological Macromolecules 222: 1110–1126. 10.1016/j.ijbiomac.2022.09.227. 36181889

[fsn370453-bib-0053] Xue, H.‐Y. , J.‐R. Li , Y.‐G. Liu , et al. 2019. “Optimization of the Ultrafiltration‐Assisted Extraction of Chinese Yam Polysaccharide Using Response Surface Methodology and Its Biological Activity.” International Journal of Biological Macromolecules 121: 1186–1193. 10.1016/j.ijbiomac.2018.10.126. 30342144

[fsn370453-bib-0054] Xue, S. , X. Hu , L. Zhu , L. Nie , and G. Li . 2019. “Protective Functions of *Lycium barbarum* Polysaccharides in H_2_O_2_‐Injured Vascular Endothelial Cells Through Anti‐Oxidation and Anti‐Apoptosis Effects.” Biomedical Reports 11, no. 5: 207–214. 31632668 10.3892/br.2019.1240 PMC6792333

[fsn370453-bib-0055] Yang, P. , Y. Zhai , Y. Ma , et al. 2022. “Gas Chromatography (GC) Fingerprinting and Immunomodulatory Activity of Polysaccharide From the Rhizome of Menispermum Dauricum DC.” PeerJ 10: e13946. 36032961 10.7717/peerj.13946 PMC9406803

[fsn370453-bib-0056] Yang, W. , J. Hao , N. Chen , and J. Li . 2023. “Development of a Joint Derivatization Protocol for the Unequivocal Identification of the Monosaccharide Composition in Four Dendrobium Polysaccharides and Free Monosaccharide by GC–MS.” Biomedical Chromatography 37, no. 12: e5743. 10.1002/bmc.5743. 37700561

[fsn370453-bib-0057] Yang, W. , Z. Yang , Y. Zou , X. Sun , and G. Huang . 2022. “Extraction and Deproteinization Process of Polysaccharide From Purple Sweet Potato.” Chemical Biology & Drug Design 99, no. 1: 111–117. 10.1111/cbdd.13935. 34407290

[fsn370453-bib-0058] Yang, X. , R. Wang , S. Zhang , et al. 2014. “Polysaccharides From Panax Japonicus C.A. Meyer and Their Antioxidant Activities.” Carbohydrate Polymers 101: 386–391. 10.1016/j.carbpol.2013.09.038. 24299787

[fsn370453-bib-0059] Yu, X.‐H. , Y. Liu , X.‐L. Wu , L.‐Z. Liu , W. Fu , and D.‐D. Song . 2017. “Isolation, Purification, Characterization and Immunostimulatory Activity of Polysaccharides Derived From American Ginseng.” Carbohydrate Polymers 156: 9–18. 10.1016/j.carbpol.2016.08.092. 27842857

[fsn370453-bib-0060] Yue, Z. , J. Li , M. Tang , T. Sun , C. Chen , and Z. Wu . 2024. “Nanozyme‐Based Clusterphene for Enhanced Electrically Catalytic Cancer Therapy.” Advanced Healthcare Materials 13, no. 9: e2303222. 10.1002/adhm.202303222. 38296257

[fsn370453-bib-0061] Zhang, F. , X. Zhang , S. Guo , et al. 2020. “An Acidic Heteropolysaccharide From Lycii Fructus: Purification, Characterization, Neurotrophic and Neuroprotective Activities In Vitro.” Carbohydrate Polymers 249: 116894. 10.1016/j.carbpol.2020.116894. 32933702

[fsn370453-bib-0062] Zhang, S. , Z. Song , L. Shi , et al. 2021. “A Dandelion Polysaccharide and Its Selenium Nanoparticles: Structure Features and Evaluation of Anti‐Tumor Activity in Zebrafish Models.” Carbohydrate Polymers 270: 118365. 10.1016/j.carbpol.2021.118365. 34364610

[fsn370453-bib-0063] Zhang, X. , Z. Liu , C. Zhong , Y. Pu , Z. Yang , and Y. Bao . 2021. “Structure Characteristics and Immunomodulatory Activities of a Polysaccharide RGRP‐1b From Radix Ginseng Rubra.” International Journal of Biological Macromolecules 189: 980–992. 10.1016/j.ijbiomac.2021.08.176. 34478797

[fsn370453-bib-0064] Zhang, Y. , Z. Chen , Z. Huang , Z. Wu , J. Xu , and K. Wang . 2019. “A Comparative Study on the Structures of *Grifola frondosa* Polysaccharides Obtained by Different Decolourization Methods and Their In Vitro Antioxidant Activities.” Food & Function 10, no. 10: 6720–6731. 10.1039/C9FO01511F. 31566196

[fsn370453-bib-0065] Zhao, G. , Y. Niu , H. Wang , et al. 2024. “Effects of Three Different Plant‐Derived Polysaccharides on Growth Performance, Immunity, Antioxidant Function, and Cecal Microbiota of Broilers.” Journal of the Science of Food and Agriculture 104, no. 2: 1020–1029. 10.1002/jsfa.12988. 37718500

[fsn370453-bib-0066] Zhao, M. , F. Kuang , Y. Zhang , and G. Lv . 2023. “Effects of Hydrolysis Condition and Detection Method on the Monosaccharide Composition Analysis of Polysaccharides From Natural Sources.” Separations 11, no. 1: 2. 10.3390/separations11010002.

[fsn370453-bib-0067] Zhao, Y. , Y. Feng , X. Jing , Y. Liu , and A. Liu . 2021. “Structural Characterization of an Alkali‐Soluble Polysaccharide From Angelica Sinensis and Its Antitumor Activity In Vivo.” Chemistry & Biodiversity 18, no. 6: e2100089. 10.1002/cbdv.202100089. 33893719

[fsn370453-bib-0068] Zhi, N. , X. Chang , X. Wang , J. Guo , J. Chen , and S. Gui . 2024. “Recent Advances in the Extraction, Purification, Structural‐Property Correlations, and Antiobesity Mechanism of Traditional Chinese Medicine‐Derived Polysaccharides: A Review.” Frontiers in Nutrition 10: 1341583. 10.3389/fnut.2023.1341583. 38299183 PMC10828026

[fsn370453-bib-0069] Zhou, S. , G. Huang , and G. Chen . 2021. “Extraction, Structural Analysis, Derivatization and Antioxidant Activity of Polysaccharide From Chinese Yam.” Food Chemistry 361: 130089. 10.1016/j.foodchem.2021.130089. 34029907

[fsn370453-bib-0070] Zhou, S. , A. Rahman , J. Li , et al. 2020. “Extraction Methods Affect the Structure of Goji (*Lycium barbarum*) Polysaccharides.” Molecules 25, no. 4: 936. 10.3390/molecules25040936. 32093113 PMC7070559

[fsn370453-bib-0071] Zhou, S. , Z. Wang , Y. Hao , P. An , J. Luo , and Y. Luo . 2023. “Dandelion Polysaccharides Ameliorate High‐Fat‐Diet‐Induced Atherosclerosis in Mice Through Antioxidant and Anti‐Inflammatory Capabilities.” Nutrients 15, no. 19: 4120. 10.3390/nu15194120. 37836404 PMC10574455

[fsn370453-bib-0072] Zhou, Y. , H. Yin , and S. Ai . 2023. “Recent Advances and Applications of Bi2S3‐Based Composites in Photoelectrochemical Sensors and Biosensors.” TrAC Trends in Analytical Chemistry 158: 116876. 10.1016/j.trac.2022.116876.

[fsn370453-bib-0073] Zhu, B. , W. Zhang , J. Zhao , B. Chen , F. Liu , and S. Li . 2022. “Characterization and Comparison of Bioactive Polysaccharides From Grifola Frondosa by HPSEC‐MALLS‐RID and Saccharide Mapping Based on HPAEC‐PAD.” Polymers 15, no. 1: 208. 10.3390/polym15010208. 36616557 PMC9824690

[fsn370453-bib-0074] Zou, Y.‐F. , C.‐Y. Li , Y.‐P. Fu , et al. 2022. “The Comparison of Preliminary Structure and Intestinal Anti‐Inflammatory and Anti‐Oxidative Activities of Polysaccharides From Different Root Parts of *Angelica sinensis* (Oliv.) Diels.” Journal of Ethnopharmacology 295: 115446. 10.1016/j.jep.2022.115446. 35675860

